# Incorporating Breast Cancer Recurrence Events Into Population-Based Cancer Registries Using Medical Claims: Cohort Study

**DOI:** 10.2196/18143

**Published:** 2020-08-17

**Authors:** Teresa A'mar, J David Beatty, Catherine Fedorenko, Daniel Markowitz, Thomas Corey, Jane Lange, Stephen M Schwartz, Bin Huang, Jessica Chubak, Ruth Etzioni

**Affiliations:** 1 Public Health Sciences Fred Hutchinson Cancer Research Center Seattle, WA United States; 2 Swedish Cancer Institute Seattle, WA United States; 3 College of Medicine University of Kentucky Lexington, KY United States; 4 Kaiser Permanente Washington Health Research Institute Seattle, WA United States

**Keywords:** cancer registries, medical claims, cancer recurrence event, statistical learning, breast cancer, medical informatics, data mining

## Abstract

**Background:**

There is a need for automated approaches to incorporate information on cancer recurrence events into population-based cancer registries.

**Objective:**

The aim of this study is to determine the accuracy of a novel data mining algorithm to extract information from linked registry and medical claims data on the occurrence and timing of second breast cancer events (SBCE).

**Methods:**

We used supervised data from 3092 stage I and II breast cancer cases (with 394 recurrences), diagnosed between 1993 and 2006 inclusive, of patients at Kaiser Permanente Washington and cases in the Puget Sound Cancer Surveillance System. Our goal was to classify each month after primary treatment as pre- versus post-SBCE. The prediction feature set for a given month consisted of registry variables on disease and patient characteristics related to the primary breast cancer event, as well as features based on monthly counts of diagnosis and procedure codes for the current, prior, and future months. A month was classified as post-SBCE if the predicted probability exceeded a probability threshold (PT); the predicted time of the SBCE was taken to be the month of maximum increase in the predicted probability between adjacent months.

**Results:**

The Kaplan-Meier net probability of SBCE was 0.25 at 14 years. The month-level receiver operating characteristic curve on test data (20% of the data set) had an area under the curve of 0.986. The person-level predictions (at a monthly PT of 0.5) had a sensitivity of 0.89, a specificity of 0.98, a positive predictive value of 0.85, and a negative predictive value of 0.98. The corresponding median difference between the observed and predicted months of recurrence was 0 and the mean difference was 0.04 months.

**Conclusions:**

Data mining of medical claims holds promise for the streamlining of cancer registry operations to feasibly collect information about second breast cancer events.

## Introduction

Population-based cancer registries are indispensable for tracking the evolving burden of cancer in the population. In the United States, the Surveillance, Epidemiology, and End Results (SEER) Program [1] of the National Cancer Institute (NCI) is a national resource for population-based information on cancer incidence, mortality, and survival. SEER provides curated, quality-controlled information on demographics, disease characteristics at diagnosis, and primary treatments for newly diagnosed patients in 18 geographically defined catchment areas around the country.

While SEER is a primary source of information about the population cancer burden, it currently focuses on primary diagnoses of cancer and the first course of treatment. Mortality information is added via annual linkages to vital status records from the National Center for Health Statistics and State Health departments. Beyond the date and cause of death, information on postdiagnosis outcomes such as cancer recurrence or progression is not collected, except for subsequent primary tumors. A prospective system for recording recurrences in the SEER registries would require expanded reporting by health care facilities and providers and the requisite financial support to extract and process the necessary information. The absence of such an infrastructure in SEER has driven efforts to harness administrative claims data for recurrence identification.

Claims-based approaches use a patient’s pattern of medical claims to identify the recurrence event at the individual level. Initial claims-based breast cancer recurrence algorithms were “clinically intuitive,” (ie, based on beliefs about what diagnosis or procedure codes would be used at the time of a recurrence) [2-5]. Recently, more automated statistical learning and data mining approaches have been harnessed to predict recurrence events from claims histories. Chubak et al [6] used classification and regression tree analysis to predict whether a patient had experienced a breast cancer recurrence or second breast cancer diagnosis. Ritzwoller et al [7] used a combination of logistic regression and changepoint detection to identify the presence and timing of recurrence events. Both of these contributions focused on identifying outcomes for research studies; in this study, we focus on a surveillance application, motivated by the lack of recurrence information in cancer registries and the consequent absence of recurrence in registry-based assessments of population disease burden.

In this article, we present a statistical learning algorithm to predict second breast cancer event (SBCE) occurrence and timing using a cancer information registry linked with medical claims among women with localized breast cancer diagnosed in the Puget Sound SEER cancer registry (Cancer Surveillance System) and treated at Kaiser Permanente Washington (KPWA), formerly Group Health. Our work differs from that of Ritzwoller et al [7] and Chubak et al [6] in several ways. First, we use a gradient boosting algorithm which generally provides improved performance over logistic regression or single trees as used in these previous studies. Our definition of the learning problem (as a month-based classification problem) and our use of gradient boosting permitted the inclusion of a large number of predictors, including some novel predictors that leveraged our learning problem definition and improved performance over the Chubak algorithm in this data set. Additionally, in contrast to prior studies which focused on research applications, our entire focus is on the augmentation of cancer registries; this guides our evaluation of predictive performance and recommendations for practical applications of our work.

## Methods

### Definitions and Overview

The standard definition of cancer recurrence is the return or rediagnosis of disease after an apparently disease-free interval. In contrast, cancer progression is any transition to a more advanced disease state without a disease-free interval. In this manuscript, we focus on SBCEs, which we define as a resurfacing of the original breast cancer (ie, recurrence) or a diagnosis of a new breast cancer. We focus on the first SBCE after the primary breast cancer diagnosis. Our goal was to use the entire record of claims for a patient to predict whether (and when) a recurrence has occurred, not to predict imminent or future recurrence for real-time clinical care, which would only be able to use claims up to the time of prediction.

Resolution of the defined prediction problem rests on the following: (1) the availability of a large enough sample of patients with claims histories and gold standard SBCE data; (2) claims histories that are adequately rich so that features predictive of SBCE can be extracted; (3) a prediction algorithm that outputs a prediction of both the presence of an SBCE within an individual patient and the timing of the event; and (4) a set of metrics for assessment of the performance of the prediction algorithm. We discuss each of these below.

### Study Population and Gold Standard

The study population was female KPWA patients aged 18 and older with a first primary, unilateral, stage I-II breast cancer between 1993 and 2006. We used Cancer Surveillance System, the SEER registry for the Puget Sound area, to identify these cases. Only patients who remained enrolled at KPWA for 1 year after their breast cancer diagnosis (unless they died) were included. Additional eligibility criteria have been described previously [6]; a total of 3152 patients were eligible.

Through structured medical record abstraction of KPWA charts (both paper and electronic), we confirmed eligibility and collected gold standard data on breast cancer recurrence and second primary breast cancers. Abstractors had access to the full medical record, which included clinician progress notes, imaging reports, surgical reports, and pathology reports. Based on this information, a recurrence was defined as an invasive tumor in the ipsilateral breast or lymph nodes, or a distant tumor, occurring at least 120 days after definitive surgery for the index breast cancer. A second breast primary was defined as a contralateral breast tumor, occurring at least 120 days after definitive surgery for the index breast cancer. Additionally, a second breast cancer in the ipsilateral breast after breast-conserving surgery is considered a second primary in SEER if it is confirmed by histological evaluation and tumor markers to be distinct from the index primary; or occurs over 5 years from the date of diagnosis of the index primary breast cancer. Chart-abstracted data were considered the gold standard in algorithm development. The KPWA Human Subjects Research Committee approved study activities.

Deidentified data only (with all dates stored as days since diagnosis of the first primary) were available for the current analysis. Patient-level data were augmented to include a randomly generated month and day of diagnosis and fractional year for age on the day of diagnosis. These changes allowed us to include the time since diagnosis and age in real numbers as month-level predictor variables, as well as summarize the claims information by calendar month.

### Predictor Variables

Candidate predictors for algorithm development included registry variables summarizing demographic (eg, age) and disease characteristics (eg, site, stage, grade, hormone receptor status) at diagnosis and variables defined on the basis of the health care utilization (henceforth called “claims,” though most codes resulted from health care within the KPWA system and not from external providers who submitted actual claims for reimbursement). Procedures and diagnoses were identified using standard coding systems (International Classification of Diseases, Ninth Revision, Clinical Modification [ICD-9-CM], Current Procedural Terminology [CPT], and Healthcare Common Procedure Coding System [HCPCS]).

Valid claims were defined to be claims after the analysis start date (6 months after the primary breast cancer diagnosis) until the end of follow-up. For patients with a nonbreast second primary cancer, the end of follow-up was set to 3 months before the registry-based diagnosis date. For patients with more than one SBCE, we included all claims before and after the event but censored the data 1 month before the first subsequent breast cancer event. For patients without an SBCE we included all claims recorded until the end of follow-up.

For each individual, we consolidated claims by days since primary breast cancer diagnosis so that any diagnosis or procedure code occurred at most once per day. Additionally, all diagnosis codes included in the analyses had to occur at least twice (ie, on two separate days) for at least one individual. Codes were then summed by calendar month for each individual to create a monthly count total for each code.

We grouped codes that were similar or that captured the same clinical condition or medical procedure type using code groupings specified in Chubak et al [6], which implemented both coarser and finer grouping systems. The coarser groups had 11 diagnostic code groups and 22 procedure groups, and the finer scale groups had 77 diagnostic code groups and 156 procedure code groups. In our analysis, we used these finer level groups (Multimedia Appendix 1).

### Prediction Problem Definition and Feature Engineering

We formulated our prediction objective as a classification problem on a person-month level. The goal was to classify months as either pre- or post-SBCE (including the month of SBCE). In this way, we transformed the problem of predicting a person-level time to event into a binary classification problem at the level of a person-month. Features used to predict the SBCE status for each month included baseline registry variables, months since diagnosis, age of the patient in the month, and a set of counts representing the number of occurrences of each code group within the month. In addition, we counted the number of months since the last occurrence of each code group as well as the number of months until the next occurrence. A default value of –1 was used when no instance of the code group was observed before or after the current month. An additional set of features consisted of the fraction of the prior months containing at least one instance of each code group.

We adopted a gradient-boosting algorithm (function XGBoost in R; R Foundation for Statistical Computing) [8] for the predictive analysis. Gradient boosting is an iterative, ensemble algorithm that incorporates multiple classification models; XGBoost is an optimized, distributed gradient boosting library designed by Chen et al [8]. The data for both the non-SBCE and SBCE patients were each split 80:20 and combined into training and test sets, respectively. The training set was split into 5 groups for cross-validation in a stratified fashion, to identify flexibility parameters that produced optimal out-of-sample performance.

### Performance Metrics

All performance metrics were calculated on the test data set. We evaluated predictive performance at both a person-month level and a person level. Person-month–level accuracy was captured via receiver operating characteristic (ROC) curves and area under the curve (AUC) statistics.

For person-level predictions, we defined a grid of threshold probabilities between 0.10 and 0.75, and defined a person as having an SBCE if any of their month-level predictions exceeded the threshold. The predicted time of an SBCE was set to be the first month for which the month-level prediction exceeded the threshold. The sensitivity and specificity of the person-level predictions were assessed along with person-level positive and negative predictive values.

We assessed the accuracy of the predicted time of SBCE by calculating the mean and median difference between the predicted and actual time of the event for persons correctly predicted to have an SBCE. We also graphed a Kaplan-Meier curve of the predicted time to an SBCE and compared it against the Kaplan-Meier curve of the observed time among all patients with an SBCE. Thus, person-level accuracy of the SBCE prediction and its timing were calculated for each threshold probability.

## Results

There were 3152 eligible patients. Baseline demographic and clinical characteristics of these cases have been previously summarized [6]. SBCE patients were more likely to have been diagnosed in an earlier calendar year, to have regional rather than localized disease, to have tumors that are not as well-differentiated, and to have negative estrogen or progesterone receptor status. The cumulative net (Kaplan-Meier–based) probability of an SBCE was 25% over 14 years follow-up.

**Figure 1:**
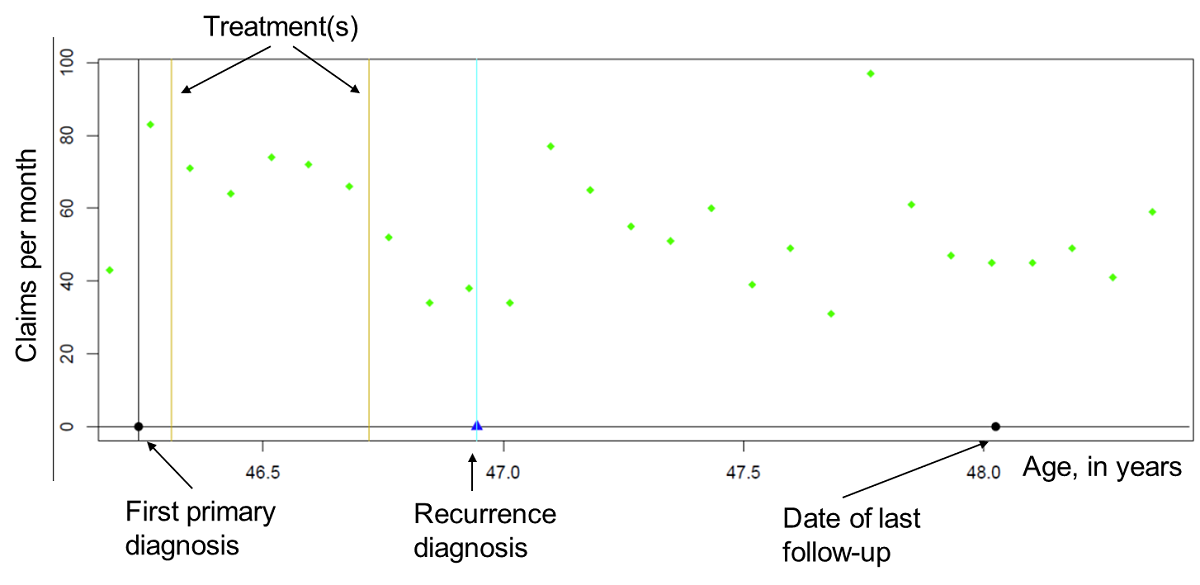
Sample plots for a hypothetical case showing a typical pattern of recorded claims each month before and after a second breast cancer event (SBCE).

Of the 3152 initially eligible patients, 3102 had at least one month of claims starting 6 months after the initial date of diagnosis up to a maximum of 159 months, 2698 without an SBCE and 404 with an SBCE. Figure 1 shows an individual-level profile of the number of claims per month for a hypothetical SBCE case.

Of the 404 patients with an SBCE, 394 had at least one month of claims after the date of the second event. Predictive analyses excluded the 10 recurrent cases with no claims after their SBCE date, yielding a final sample size of 3092 patients. In the monthly claims data for the 3092 patients included in the analysis, there were 543 unique diagnostic codes and 992 unique procedure codes.

The training set included monthly claims data for 2160 patients without an SBCE and 315 with an SBCE. The test set included monthly claims data for 538 patients without an SBCE and 79 with an SBCE.

The number of months of available claims was slightly longer in patients with an SBCE (range 3-138, mean 44.7, median 39.5, SD 26.2 months) compared to those without an SBCE (range 1-149, mean 31.5, median 28, SD 19.2 months). SBCE cases had claims for a median of 21.9 months before and 19 months after the SBCE.

Table 1 displays the 20 features with highest importance identified by the gradient boosting algorithm. The features with highest importance are those most commonly present in the submodels that constitute the final algorithm. They primarily include secondary malignancy, imaging tests, diagnostic tests, and salvage treatments.

**Table 1:** Top 20 features identified by the gradient boosting algorithm.

Order	Description
1	Fraction of prior months with diagnosis code for secondary malignant neoplasm of other specified sites
2	Fraction of prior months with procedure codes for biopsy or excision of lymph nodes
3	Months since last procedure code for needle biopsy
4	Fraction of prior months with diagnosis codes for secondary malignant neoplasm of respiratory and digestive systems
5	Months since last procedure code for bone scan
6	Months since last procedure code for other tumor markers
7	Months since last diagnosis code for carcinoma in situ of breast and genitourinary system
8	Fraction of prior months with diagnosis code for cancer of breast
9	Time until next diagnosis code for secondary malignant neoplasm of respiratory and digestive systems
10	Fraction of prior months with procedure code for fine needle aspirate
11	Number of instances of diagnosis code for cancer of breast in the current month
12	Months since procedure code for biopsy or excision of lymph nodes
13	Fraction of prior months with procedure code for chemotherapy
14	Months since diagnosis
15	Months since last procedure code for chest computed tomography
16	Fraction of prior months with procedure code for bone scan
17	Age in current month
18	Fraction of prior months with diagnosis code for benign mammary dysplasias
19	Time until next diagnosis code for secondary malignant neoplasm of other specified sites
20	Time until next diagnosis code for cancer of other and unspecified sites

**Figure 2:**
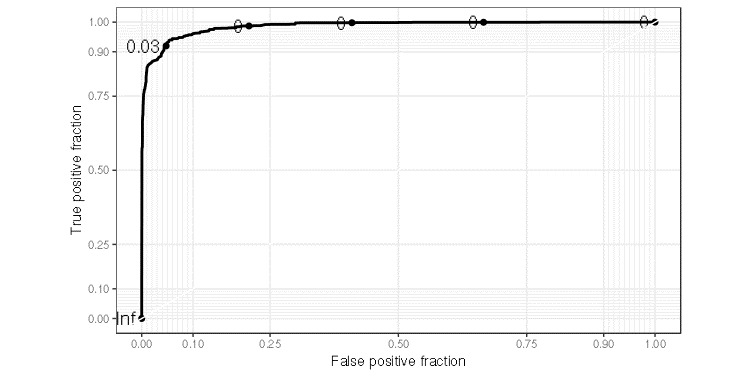
Month-level receiver operating characteristic (ROC) curve based on the test data set corresponding to the prediction model derived using the training data set. The area under the curve (AUC) is 0.986.

The AUC for month-level ROC curve in the test data set was 0.986 (Figure 2). Multimedia Appendix 2 shows the monthly SBCE status (0 for pre-SBCE; 1 for post-SBCE, including month of SBCE), along with predicted probabilities of being post-SBCE for a randomly selected set of 12 non-SBCE cases in the test set; Multimedia Appendix 3 presents similar results for 12 SBCE cases. The predicted probabilities generally tracked well with the observed outcomes, but performance in SBCE cases degraded over time in some cases after the month of the event.

Table 2 provides the person-level performance for various thresholds for classifying an individual as having an SBCE. For each threshold, an individual was classified as having an SBCE if at least one of the monthly predicted probabilities (of being post-SBCE) exceeds the threshold. Lower thresholds are associated with greater sensitivity but lower specificity and positive predictive value (PPV). The sensitivity, specificity, PPV, and negative predictive value (NPV) corresponding to a threshold of 0.5 are 88.6%, 97.8%, 85.4%, and 98.3% respectively. As the threshold increases, the PPV improves, and as the threshold decreases, the NPV improves.

**Table 2:** Person-level performance (sensitivity, specificity, and positive and negative predictive values) corresponding to various probability thresholds for classifying an individual as having a second breast cancer event^a^.

Threshold	Sensitivity	Specificity	Positive predictive value	Negative predictive value
0.10	0.962	0.942	0.710	0.994
0.15	0.937	0.950	0.733	0.990
0.20	0.924	0.955	0.753	0.988
0.25	0.911	0.957	0.758	0.987
0.30	0.886	0.959	0.761	0.983
0.35	0.886	0.963	0.778	0.983
0.40	0.886	0.972	0.824	0.983
0.45	0.886	0.976	0.843	0.983
0.50	0.886	0.978	0.854	0.983
0.55	0.886	0.980	0.864	0.983
0.60	0.886	0.980	0.864	0.983
0.65	0.886	0.981	0.875	0.983
0.70	0.873	0.983	0.885	0.981
0.75	0.861	0.987	0.907	0.980

^a^For each threshold, an individual is predicted to have a second breast cancer event if at least one of the monthly predicted probabilities exceeds the threshold. There were 538 cases without and 79 cases with a second breast cancer event in the test set.

Table 3 summarizes the accuracy of the predicted timing of SBCE at each threshold probability. For a threshold of 0.5, the mean difference in months between the predicted and observed month of recurrence for correctly classified recurrent cases is 0.04 months (SD 3.5 months) and the median difference is zero.

**Figure 3:**
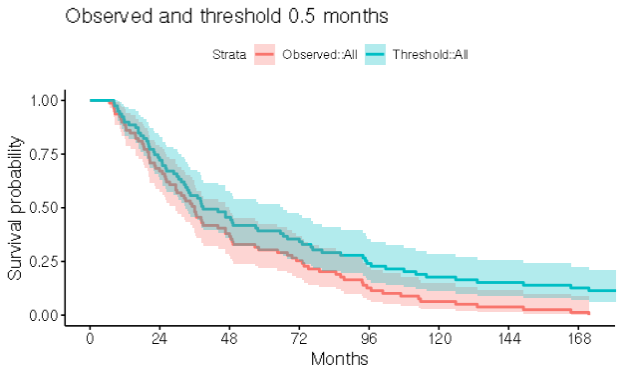
Accuracy of predicted timing of recurrence expressed via a comparison of Kaplan-Meier curves for observed (red) versus predicted (blue) time to SBCE among test set cases with a SBCE, where the predicted time to SBCE is based on a threshold probability of 0.5. Cases for whom no SBCE is predicted (monthly predicted probabilities never exceed 0.5) are censored at their last follow-up time. SBCE: second breast cancer event.

Figure 3 plots a Kaplan-Meier curve of the observed time to SBCE among SBCE cases in the test data set, overlaid with a similar curve of the predicted time to SBCE (defined as the first month for which the predicted probability of being post-SBCE exceeds 0.5). In the predicted curve, cases for which no SBCE is predicted are censored at their last follow-up time. The observed and predicted curves confirm the favorable performance of the prediction algorithm in terms of both person-level diagnostic performance and timing. Note that these results may vary slightly depending on the random number seed/initialization used to split the data into the training and test sets and perform the cross-validation subselection used in the XGBoost algorithm.

**Table 3:** Accuracy of the predicted timing of a second breast cancer event at each of a set of threshold probabilities^a^.

Threshold	Predicted number of second breast cancer events	Mean difference in months	Median difference in months	Minimum difference in months	Maximum difference in months
0.10	76	–1.5	0	–36	19
0.15	74	–0.8	0	–27	19
0.20	73	–0.3	0	–24	19
0.25	72	–0.3	0	–24	19
0.30	70	–0.5	0	–24	5
0.35	70	–0.5	0	–24	5
0.40	70	–0.3	0	–24	5
0.45	70	–0.2	0	–24	5
0.50	70	–0.04	0	–24	5
0.55	70	0.01	0	–24	5
0.60	70	0.1	0	–24	5
0.65	70	0.1	0	–24	5
0.70	69	0.3	0	–24	9
0.75	68	0.4	0	–24	9

^a^The table shows the mean, median, maximum, and minimum of the difference between the observed and predicted time of a second breast cancer event given the threshold for each of the individuals correctly predicted to have a second breast cancer event. A negative value indicates that the predicted time of a second breast cancer event precedes the observed time. For each threshold, an individual is determined to have had a second breast cancer event if at least one of the monthly predicted probabilities exceeds the threshold. There are 79 individuals with a second breast cancer event in the test data.

## Discussion

This study tackles the overarching question of how best to harness electronic health data to inform cancer registries about disease recurrence events and to augment them to add this information. The core of our contribution centers on data mining of medical claims histories using a relatively established gradient boosting algorithm. The algorithm and the accompanying features expand on and complement published data mining approaches that use claims histories to learn about the risk of disease recurrence. Furthermore, our focus on surveillance, which drives our learning problem definition, performance evaluation, and recommendation, differs from existing work that focuses on clinical prognostication.

Our approach yields a continuous prediction per each valid claims month, to which a threshold can be applied to yield a level of diagnostic performance that is most consistent with a prespecified performance. A higher threshold raises sensitivity and lowers specificity. A lower threshold has the opposite effect. If achieving high NPV is the primary objective, then a lower threshold might potentially be preferred. With a NPV of 99.4% at a changepoint threshold of 0.10, our algorithm could be offered to registries as a tool for ruling out an SBCE [9]. Indeed, in the test data set with sample size of 617, a threshold of 0.10 classified 510 individuals as not having an SBCE. Therefore, if an NPV of 99.4% was deemed to be adequate, use of the algorithm would mean that the registry could focus recurrence-identification resources on 17% (107/617) of the case population.

Our approach has one feature in common with that of Ritzwoller et al [7], who predicted cancer recurrence based on medical claims among cases with lung and colorectal cancer. Their two-step procedure first predicted individual-level recurrence status and then predicted its timing by identifying the month of greatest change in the count of each code grouping, and reconciling the months so identified across the groupings. Our procedure merges the prediction of the presence of recurrence and the timing of recurrence, and applies a similar changepoint idea, but to the single series of monthly predicted probabilities of being post recurrence. This avoids the need to reconcile different predictions, and accommodates a large number of novel features that leverage the month-based definition of the statistical learning problem.

Any method that uses medical claims to predict SBCE status will ideally require continuous and complete claims histories on all registry cases. In practice, there are likely to be gaps in coverage and some claims histories may be partially missing. Further, claims histories will not be available for uninsured cases, limiting the representativeness of the population for which recurrence information will be made available via our approach. In the KPWA data used here, most patients retained health system coverage over time, reducing the extent of this problem in the current analysis.

We foresee offering this algorithm as part of population-based center cancer registries’ data capturing process. One critical reason that recurrence data are not well captured is that abstractors do not have enough time to look over all cancer cases periodically to identify any recurrence. Utilizing our algorithm, a subset of probable recurrences can be marked for further abstraction to verify the occurrence and timing of a recurrence. The threshold can be adjusted based on the resources available in the individual registry.

There are other limitations that arise from reliance on medical claims data as an approach for augmenting cancer registries. Diagnosis and procedure coding systems change over time and so claims-based algorithms will need frequent review and updating to remain current [10]. Even for those patients who are insured, gaps in coverage will inevitably arise as patients lose coverage or transition between insurance plans. Some insurance plans may not agree to participate in a linkage with the cancer registry. In any registry catchment area, there will be multiple payers; agreements will have to be executed with all of them for maximum coverage and linkages across plans will have to be implemented. These logistical issues are important but secondary to the critical first step showing that the linkages are likely to provide valid, useful, and useable information to inform health care professionals about disease recurrence. Further work is ongoing to investigate how the performance of our data-mining approach transfers to a setting in which there are multiple payers and coverage gaps or nonuniversal availability of claims linkages within a registry catchment area.

## Appendix

Multimedia Appendix 1 Diagnostic and procedure code groups and types.

Multimedia Appendix 2 Monthly SBCE status along with predicted probabilities of being post-SBCE for a randomly selected set of 12 non-SBCE cases in the test set.

Multimedia Appendix 3 Monthly SBCE status along with predicted probabilities of being post-SBCE for a randomly selected set of 12 cases with an SBCE in the test set.

## Abbreviations

AUC: area under the curve
KPWA: Kaiser Permanente Washington
NCI: National Cancer Institute
NPV: negative predictive value
PPV: positive predictive value
PT: probability threshold
ROC: receiver operating characteristic
SBCE: second breast cancer events
SEER: Surveillance, Epidemiology and End Results
