# Correction: Incorporating Breast Cancer Recurrence Events Into Population-Based Cancer Registries Using Medical Claims: Cohort Study

**DOI:** 10.2196/23821

**Published:** 2020-09-24

**Authors:** Teresa A'mar, J David Beatty, Catherine Fedorenko, Daniel Markowitz, Thomas Corey, Jane Lange, Stephen M Schwartz, Bin Huang, Jessica Chubak, Ruth Etzioni

**Affiliations:** 1 Public Health Sciences Fred Hutchinson Cancer Research Center Seattle, WA United States; 2 Swedish Cancer Institute Seattle, WA United States; 3 College of Medicine University of Kentucky Lexington, KY United States; 4 Kaiser Permanente Washington Health Research Institute Seattle, WA United States

In “Incorporating Breast Cancer Recurrence Events Into Population-Based Cancer Registries Using Medical Claims: Cohort Study" (JMIR Cancer 2020;6(2):e18143) the authors noted two errors.

The metadata erroneously listed only Teresa A'mar and Ruth Etzioni as having contributed equally; this has been corrected to reflect that Teresa A'mar, Jessica Chubak, and Ruth Etzioni contributed equally.

In addition, Jessica Chubak's affiliation was originally listed as:

Washington Health Research Institute, Kaiser Permanente, Seattle, WA, United States

This affiliation has been corrected to:

Kaiser Permanente Washington Health Research Institute, Seattle, WA, United States

The correction will appear in the online version of the paper on the JMIR website on September 24, 2020, toegether with the publication of this correction notice. Because this was made after submission to PubMed, PubMed Central, and other full-text repositories, the corrected article has also been submitted to those repositories.

