# Supplementary material for: Incorporating Breast Cancer Recurrence Events Into Population-Based Cancer Registries Using Medical Claims: Cohort Study
Source: JMIR Cancer. 2020 Aug 17;6(2):e18143. doi: 10.2196/18143 (PMC7459434; doi:10.2196/18143)
Supplement: Multimedia Appendix 1 [file cancer_v6i2e18143_app1.docx]

Multimedia Appendix 1. Diagnostic and procedure code groups and types; 11 diagnostic categories and 77 diagnostic types; 22 procedure categories and 156 procedure types.

| **Category** | **Type** |
| --- | --- |
| Cancer | Cancer of bone and connective tissue |
| Cancer | Cancer of breast |
| Cancer | Cancer of genitourinary organs |
| Cancer | Cancer of lip, oral cavity, and pharynx |
| Cancer | Cancer of lymphatic and hematopoietic tissue |
| Cancer | Cancer of other and unspecified sites |
| Cancer | Cancer of respiratory and intrathoracic organs |
| Cancer | Cancer of the digestive organs and peritoneum |
| Cancer | Carcinoma in situ of breast |
| Cancer | Carcinoma in situ of breast and genitourinary system |
| Cancer | Carcinoma in situ of digestive organs |
| Cancer | Carcinoma in situ of genitourinary system |
| Cancer | Carcinoma in situ of other and unspecified sites |
| Cancer | Carcinoma in situ of respiratory system |
| Cancer | Carcinoma in situ of skin |
| Cancer | Neoplasm of uncertain behavior of degestive and respiratory system |
| Cancer | Neoplasm of uncertain behavior of endcrine glands and nervous system |
| Cancer | Neoplasm of uncertain behavior of genitourinary system |
| Cancer | Neoplasm of uncertain behavior of other an unspecifed sites and tissues |
| Cancer | Neoplasms of uncertain behavior |
| Cancer | Neoplasms of unspecified nature |
| Clinical signs | Abdominal or pelvic swelling, mass, or lump |
| Clinical signs | Abdominal pain |
| Clinical signs | Abdominal rigidity |
| Clinical signs | Abdominal tenderness |
| Clinical signs | Ascites |
| Clinical signs | Enlargement of liver |
| Clinical signs | Enlargement of spleen |
| Clinical signs | Lymphedema |
| Clinical signs | Neurologic abnormalities |
| Clinical signs | Pain in other locations (than the breast) |
| Clinical signs | Skin changes, nodules |
| Clinical signs | Unspecified liver disorder |
| Imaging of breast or suspected site(s) of recurrence | Bone scan misc code |
| Imaging of breast or suspected site(s) of recurrence | Imaging using other radiologic or nuclear medicine - site(s) of recurrence |
| Imaging of breast or suspected site(s) of recurrence | Mammography |
| Laboratory Tests | Abnormal Blood |
| Laboratory Tests | Laboratory Tests |
| Metastasis or unknown behavior | Malignant neoplasm without specification of site |
| Metastasis or unknown behavior | Secondary and unspecified malignant neoplasm of lymph nodes |
| Metastasis or unknown behavior | Secondary malignant neoplasm of other specified sites |
| Metastasis or unknown behavior | Secondary malignant neoplasm of respiratory and digestive systems |
| Ovarian Ablation | Surgical ovarian ablation |
| Pathology | Other tumor markers |
| Patient symptoms | Abdominal pain |
| Patient symptoms | Adenopathy |
| Patient symptoms | Benign mammary dysplasias |
| Patient symptoms | Bleeding |
| Patient symptoms | Brachial plexopathy |
| Patient symptoms | Laboratory Tests |
| Patient symptoms | Low blood pressure |
| Patient symptoms | Lymphedema |
| Patient symptoms | Mass in breast, nipple change or breast pain |
| Patient symptoms | Other disorders of breast |
| Patient symptoms | Pain |
| Patient symptoms | Pain due to cancer |
| Patient symptoms | Pain in other locations (than the breast) |
| Patient symptoms | Pathologic fractures |
| Patient symptoms | Weakness |
| Patient symptoms | Weight loss |
| Staging | Abnormal Blood |
| Staging | Bone scan misc code |
| Staging | Imaging using other radiologic or nuclear medicine - site(s) of recurrence |
| Staging | Laboratory Tests |
| Staging | Mammography |
| Supportive/Palliative care | Alterations in consciousness |
| Supportive/Palliative care | Anemia |
| Supportive/Palliative care | Bleeding |
| Supportive/Palliative care | Blood in stool |
| Supportive/Palliative care | Dermatitis |
| Supportive/Palliative care | Difficulty swallowing |
| Supportive/Palliative care | Fever |
| Supportive/Palliative care | Gastrointestinal disease |
| Supportive/Palliative care | General symptoms |
| Supportive/Palliative care | Infection |
| Supportive/Palliative care | Inflammation or disease of pericardium |
| Supportive/Palliative care | Nausea or vomitting |
| Supportive/Palliative care | Nutrition |
| Supportive/Palliative care | Pleural effusions |
| Supportive/Palliative care | Pleurisy |
| Supportive/Palliative care | Pressure ulcer |
| Supportive/Palliative care | Psychiatric |
| Supportive/Palliative care | Respiratory disease |
| Supportive/Palliative care | Weight loss |
| Systemic Therapy | Anemia from chemotherapy |
| Systemic Therapy | Chemotherapy |
| Systemic Therapy | Chemotherapy misc |
| Systemic Therapy | Neutropenia from chemotherapy |

| **Category** | **Type** |
| --- | --- |
| Bone Metastases | Bisphosphonates |
| Breast biopsy | Fine needle aspirate |
| Breast biopsy | Needle biopsy |
| Breast biopsy | Needle biopsy misc code |
| Breast biopsy | Other breast biopsy |
| Breast biopsy | Ultrasound - site(s) of recurrence |
| Breast surgery | Anesthesia for breast surgery |
| Breast surgery | Breast reconstruction |
| Breast surgery | Lumpectomy |
| Breast surgery | Lumpectomy misc |
| Breast surgery | Mastectomy |
| Cancer | Breast cancer AJCC stage |
| Cancer | Breast cancer HCPC diagnosis extent of disease unknown |
| Cancer | Breast cancer HCPC diagnosis with metastases |
| Cancer | Breast cancer HCPC diagnosis with no metastases |
| Cancer | Personal history of breast cancer |
| Chest surgery | Chest surgery |
| Clinical Exams | Breast Exam |
| Clinical Exams | Follow-up exam for combined treatment |
| Clinical Exams | Follow-up exam, unspecfied |
| Clinical Exams | Mammography |
| Clinical Exams | Miscellaneous surgery codes |
| Clinical Exams | Observation for suspected malignant neoplasm |
| Clinical Exams | Other breast imaging |
| Clinical Exams | Pelvic Exam |
| Clinical Exams | Physical Exam |
| Clinical Exams | Pre-operative Exam |
| Clinical Exams | Special screening for malignant neoplasms (non-breast) |
| Clinical signs | Ascites |
| Imaging of breast or suspected site(s) of recurrence | Anesthesia for imaging |
| Imaging of breast or suspected site(s) of recurrence | Bone scan |
| Imaging of breast or suspected site(s) of recurrence | Bone scan misc code |
| Imaging of breast or suspected site(s) of recurrence | CT - chest |
| Imaging of breast or suspected site(s) of recurrence | CT - site(s) of recurrence |
| Imaging of breast or suspected site(s) of recurrence | Hybrid PET/CT - site(s) of recurrence |
| Imaging of breast or suspected site(s) of recurrence | Imaging using other radiologic or nuclear medicine - site(s) of recurrence |
| Imaging of breast or suspected site(s) of recurrence | Lymph node imaging |
| Imaging of breast or suspected site(s) of recurrence | Mammography |
| Imaging of breast or suspected site(s) of recurrence | Mammography misc |
| Imaging of breast or suspected site(s) of recurrence | Miscellaneous imaging codes- site(s) of recurrence |
| Imaging of breast or suspected site(s) of recurrence | MRI - chest |
| Imaging of breast or suspected site(s) of recurrence | MRI - site(s) of recurrence |
| Imaging of breast or suspected site(s) of recurrence | MRI of breast |
| Imaging of breast or suspected site(s) of recurrence | MRI of breast misc code |
| Imaging of breast or suspected site(s) of recurrence | Needle biopsy misc code |
| Imaging of breast or suspected site(s) of recurrence | Other breast imaging |
| Imaging of breast or suspected site(s) of recurrence | Pericardial effusions |
| Imaging of breast or suspected site(s) of recurrence | PET - site(s) of recurrence |
| Imaging of breast or suspected site(s) of recurrence | PET for breast cancer staging |
| Imaging of breast or suspected site(s) of recurrence | Radiation |
| Imaging of breast or suspected site(s) of recurrence | Scintimammography |
| Imaging of breast or suspected site(s) of recurrence | Tumor imaging |
| Imaging of breast or suspected site(s) of recurrence | Ultrasound - chest |
| Imaging of breast or suspected site(s) of recurrence | Ultrasound - site(s) of recurrence |
| Imaging of breast or suspected site(s) of recurrence | Ultrasound of breast |
| Imaging of breast or suspected site(s) of recurrence | X-ray - chest |
| Imaging of breast or suspected site(s) of recurrence | X-ray - site(s) of recurrence |
| Imaging of breast or suspected site(s) of recurrence | X-ray of breast specimen |
| Imaging of breast or suspected site(s) of recurrence | X-ray of mammary duct |
| Laboratory Tests | Hemoglobin |
| Laboratory Tests | Hormone |
| Laboratory Tests | Laboratory Tests |
| Laboratory Tests | Metabolic Test |
| Laboratory Tests | Transition Panel |
| Lymph node surgery | Biopsy or excision of lymph nodes |
| Metastasis or unknown behavior | Any metastatic disease |
| Miscellaneous surgery codes | Miscellaneous imaging codes- site(s) of recurrence |
| Miscellaneous surgery codes | Miscellaneous surgery codes |
| Other | Hyperthermia |
| Other | Photodynamic therapy |
| Other surgeries | Brain surgery |
| Other surgeries | Excision of bone lesion |
| Other surgeries | Excision of liver lesion |
| Other surgeries | Excision of lung lesion |
| Other surgeries | Pericardial effusions |
| Other surgeries | Pleural effusions |
| Other surgeries | Removal of part of lung |
| Other surgeries | Vertebroplasty or kyphoplasty |
| Ovarian Ablation | Needle biopsy misc code |
| Ovarian Ablation | Surgical ovarian ablation |
| Pathology | BRCA1/2 |
| Pathology | Circulating tumor cell test |
| Pathology | Gene expression profile for breast cancer treatment |
| Pathology | HER-2 |
| Pathology | Hormone receptor status |
| Pathology | Other Pathology |
| Pathology | Other tumor markers |
| Pathology | Pathology Findings |
| Pathology | Radiation |
| Pathology | Tissue exam by pathologist |
| Patient symptoms | Lymphedema |
| Patient symptoms | Pain |
| Patient symptoms | Weight loss |
| Pharmacy | Pharmacist |
| Radiation | Post Radiotherapy |
| Radiation | Radiation |
| Radiation | Radiopharmaceutical therapy |
| Staging | Abdominal mass biopsy or diagnostic procedure |
| Staging | Anesthesia for imaging |
| Staging | Biopsy or diagnostic procedure of ovary |
| Staging | Biopsy or diagnostic procedure on endometrium |
| Staging | Biopsy or excision of lymph nodes |
| Staging | Bone biopsy |
| Staging | Bone scan |
| Staging | Bone scan misc code |
| Staging | Brain biopsy or diagnostic procedure |
| Staging | Brain biopsy or diagnostic procedure misc code |
| Staging | CT - chest |
| Staging | CT - site(s) of recurrence |
| Staging | Hemoglobin |
| Staging | Hormone |
| Staging | Hybrid PET/CT - site(s) of recurrence |
| Staging | Imaging using other radiologic or nuclear medicine - site(s) of recurrence |
| Staging | Intestinal biopsy |
| Staging | Laboratory Tests |
| Staging | Liver biopsy or diagnostic procedure |
| Staging | Lung or chest biopsy or diagnostic procedure |
| Staging | Lymph node imaging |
| Staging | Mammography |
| Staging | Mammography misc |
| Staging | Metabolic Test |
| Staging | Microscopic examination of lymph node and lymph |
| Staging | Miscellaneous imaging codes- site(s) of recurrence |
| Staging | MRI - chest |
| Staging | MRI - site(s) of recurrence |
| Staging | MRI of breast |
| Staging | MRI of breast misc code |
| Staging | Needle biopsy misc code |
| Staging | Other breast imaging |
| Staging | Pericardial effusions |
| Staging | PET - site(s) of recurrence |
| Staging | PET for breast cancer staging |
| Staging | Radiation |
| Staging | Scintimammography |
| Staging | Tumor imaging |
| Staging | Ultrasound - chest |
| Staging | Ultrasound - site(s) of recurrence |
| Staging | Ultrasound of breast |
| Staging | X-ray - chest |
| Staging | X-ray - site(s) of recurrence |
| Staging | X-ray of breast specimen |
| Staging | X-ray of mammary duct |
| Supportive/Palliative care | Advance care planning |
| Supportive/Palliative care | Anemia |
| Supportive/Palliative care | Assistance with daily living |
| Supportive/Palliative care | Back pain |
| Supportive/Palliative care | Basic life or disability exam |
| Supportive/Palliative care | Blood pressure |
| Supportive/Palliative care | Breathing assistance |
| Supportive/Palliative care | Breathing measurements |
| Supportive/Palliative care | Collection of blood |
| Supportive/Palliative care | Convalescence and palliative care |
| Supportive/Palliative care | Dyspnea |
| Supportive/Palliative care | Emergency dept |
| Supportive/Palliative care | Erythropoiesis therapy |
| Supportive/Palliative care | Evaluation of critically ill patient |
| Supportive/Palliative care | Feeding tube |
| Supportive/Palliative care | FEV exam |
| Supportive/Palliative care | Hemoglobin exam |
| Supportive/Palliative care | Home infusion for anemia |
| Supportive/Palliative care | Home infusion for hydration |
| Supportive/Palliative care | Home infusion for nutrition |
| Supportive/Palliative care | Home infusion of drugs |
| Supportive/Palliative care | Home or hospice health service |
| Supportive/Palliative care | Home pain management |
| Supportive/Palliative care | Hospital care |
| Supportive/Palliative care | Hydration status |
| Supportive/Palliative care | Injection for therapy / diagnosis / prophylaxis |
| Supportive/Palliative care | Introduction of needle |
| Supportive/Palliative care | IV infusion for hydration |
| Supportive/Palliative care | IV infusion for therapy / diagnosis / prophylaxis |
| Supportive/Palliative care | Mental status exam |
| Supportive/Palliative care | Microalbuminuria exam |
| Supportive/Palliative care | Nursing home care |
| Supportive/Palliative care | Nutrition |
| Supportive/Palliative care | Oncology treatment |
| Supportive/Palliative care | Oxygen saturation exam |
| Supportive/Palliative care | Pain management at home |
| Supportive/Palliative care | Palliative care |
| Supportive/Palliative care | Physician home visit |
| Supportive/Palliative care | Physician on-call |
| Supportive/Palliative care | Post chemo |
| Supportive/Palliative care | Post Radiotherapy |
| Supportive/Palliative care | Prolongued physician service |
| Supportive/Palliative care | Psychiatric |
| Supportive/Palliative care | Rest home care |
| Supportive/Palliative care | Sedation |
| Supportive/Palliative care | Subcutaneous infusion |
| Supportive/Palliative care | Team conference |
| Supportive/Palliative care | Weight loss |
| Systemic Therapy | Anemia from chemotherapy |
| Systemic Therapy | Chemotherapy |
| Systemic Therapy | Chemotherapy misc |
| Systemic Therapy | Endocrine therapy |
| Systemic Therapy | Estrogen |
| Systemic Therapy | Goserelin/leuprolide |
| Systemic Therapy | Megestrol |
| Systemic Therapy | Nandrolone |
| Systemic Therapy | Pamidronate |
| Systemic Therapy | Post chemo |
| Systemic Therapy | Prednisone |
| Systemic Therapy | Targeted therapy |
| Systemic Therapy | Testosterone |
| Systemic Therapy | Treatment for nausea from chemotherapy |
