# Supplementary material for: Incorporating Breast Cancer Recurrence Events Into Population-Based Cancer Registries Using Medical Claims: Cohort Study
Source: JMIR Cancer. 2020 Aug 17;6(2):e18143. doi: 10.2196/18143 (PMC7459434; doi:10.2196/18143)

Multimedia Appendix 3. Monthly SBCE status in blue (0 for pre-SBCE; 1 for post-SBCE, including month of SBCE) along with predicted probabilities of being post-SBCE (red) for a randomly selected set of 12 cases with a SBCE in the test set.
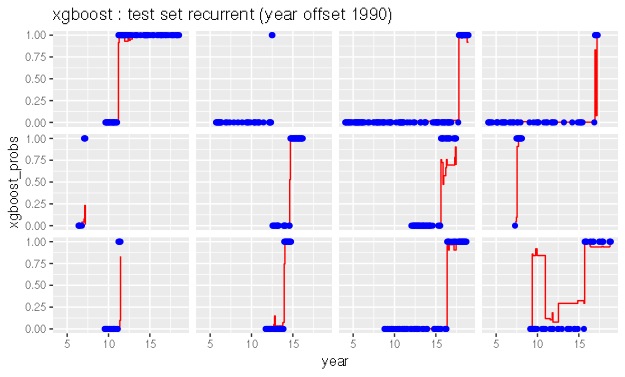

Supplement: Multimedia Appendix 3 [file cancer_v6i2e18143_app3.docx]
